# Notes on the Treatment of Acute Coryza

**Published:** 1889-01

**Authors:** Frank Hamilton Potter

**Affiliations:** Buffalo, N. Y., Lecturer on Laryngology, Medical Department, Niagara University; 273 Franklin Street


### *NOTES ON THE TREATMENT OF ACUTE CORYZA.*[1]

BY FRANK HAMILTON POTTER, M. D, Buffalo, N. Y.,

Lecturer on Laryngology, Medical Department, Niagara University.

This subject perforce divides itself at once into (1) Preventive Measures, and (2) Remedial Measures. We will consider the preventive measures first. It may be safely asserted that the

(1) Read before the Buffalo Medical and Surgical Association.

majority of colds need not occur, even in an uncertain and changing climate like ours, if people would understand and practice a few simple hygienic precautions. We cannot expect to discuss every hygienic rule in detail that would apply to each particular case, but we hope to present certain general principles which can be elaborated or reduced according to the needs of the individual.

Your attention is first directed to a custom, that may be called almost a natural one, and which we consider to be very pernicious. I refer to the habit of sleeping with the windows open at night when the external temperature is below a certain point. This custom is practically universal in this country, and is an important factor in accounting for the frequency among us of the catarrhal diseases of the upper air-passages. I fail to find, either by observation or investigation, that any other people living in our latitude indulge in this custom. The people of Europe are very careful to prevent the external night air from entering their sleeping apartments. Their windows are double, and usually you will find a closed stove or fire-box in the same room; and, in many other ways, their houses are not as well ventilated as ours.

We can derive many hints from the customs of the lower animals upon this subject. They seek to render the air, when sleeping, as warm as possible in various ways. When the temperature is below that of their bodies, they seek their dens, huddle together, or curl up so that the nose is placed close to the body. The birds even, whose native element is the air, when sleeping hide their heads beneath their wings or under their breasts. Instinctively they seem to appreciate that the body is in a state of least resistance during sleep, and that the air they then breathe should be properly warmed so as not to act as an irritant to the delicate lung structure.

Besides the suggestion just made, the reasons for greater care at night or during sleep are not difficult to understand. We remove our warm clothing and put on that which is much lighter. We are not sure on retiring that the temperature will not be twenty degrees, or more, lower in the morning. A slight restlessness will expose a part of the body to rapid cooling, which generally leads to the development of a cold.

Again, the air must reach the lungs sufficiently moist and warm, or else it will act as an irritant and cause trouble. The nose is the organ where this is accomplished, but it is limited in its ability, and cannot raise the temperature of the air beyond a certain point. According to the experiments of Aschenbrandt,[1] and Kayser,[2] the inspired air in passing through the nose can be raised about 40° F., but no more. These experiments are quoted at length by Bosworth,[3] as confirmatory of his views on the physiology of the nose, first stated in a paper read before the American Climatological Association in 1885, and entitled "Hay Fever, Asthma, and Allied Affections."

It is, of course, not unreasonable to conclude that when all the vital processes are in a state of least activity, as, during sleep, the inspired air would not be raised even as many as forty degrees, though no experiments have been made in demonstration of this. Formulating a rule, however, on the basis of these facts, we can say that when the external temperature is below 65° F. at nine o'clock in the evening, the windows of all sleeping apartments should be closed. This is the advice I have been accustomed to give patients for some time past, and it certainly has been a factor not only in relieving the chronic conditions, but in the prevention of fresh acute attacks of inflammation of the upper air passages. They will tell you they are much more comfortable on rising in the morning; the throat is less rough; there is less cough and less discharge. I believe that if this rule were universally followed, "catarrh" would not be as frequent as it is. This part of the question may be concluded with a quotation from a paper by Dr. J. B. Johnson,[4] of Washington, on this subject: "Even the most robust, who sleep in the open air, frequently awake in the morning with a husky voice, dry nostrils, pains in the limbs, and uncomfortable feelings about the chest, to tell them that they took cold during the night, and to

(1) Ueber der Bedeutung der Nase im Respiration, Th. Aschenbrandt, Wurzburg.

(2) *Pflüger's Archives*, vol. xli., pp. 127–47. 1887.

(3) *The Medical News*, August 4, 1888.

(4) The *Medical and Surgical Reporter*, August 21, 1886.

warn them against the risk and imprudence of letting the external air too freely into their sleeping-rooms at night."

Another hygienic measure of importance, especially to those who are catarrhally disposed, is the taking of a cold bath the first thing on rising in the morning. The room in which the bath is taken should be warm, and if a bath-room is convenient, the water should be drawn and allowed to stand in the tub over night. It should be taken rapidly, so that the whole body will glow afterwards. It acts as a tonic to the nervous system through the medium of the cutaneous nerves; and it serves, especially, to toughen such sensitive areas as the feet, the back of the neck, and between the shoulders. Occasionally we find a person unable to bear the slight shock of the cold water, and in that case it must be abandoned.

The subject of the proper amount of clothing is an important one, but without discussing each article in detail, it will suffice to call attention to the general rule in regard to it, viz., no part of the body should be under-clothed or over-clothed. In our climate, this requires some little care and forethought. It is manifestly improper to wear a sealskin sacque, and at the same time cover the feet merely with silk stockings and thin shoes, and expect to avoid taking cold. It is said that many deaths can be attributed every year to the sealskin sacque. Its wearers seldom remove it upon going indoors on their social visits, and have been known to remain in an over-heated room for an hour or more with it on, thus producing more or less perspiration and consequent rapid chilling of some surface of the body on returning to the outside air. That the outer wraps should always be removed upon going indoors, is like stating a truism, but how often do you see it violated not only by the wearers of sealskin sacques, but by the wearers of overcoats as well!

Another article may require a few words. The neck-scarf is generally considered unnecessary and dangerous, and we find much advice against its use. It would seem, however, that it is the abuse, not the use, of it that is dangerous. There are many people to whom it is a necessity, and the danger to them consists in not wearing it. They should be advised that it should be worn according to the temperature of the day. On the sudden warm days of winter, when it would produce perspiration, it may be turned back or carried in the pocket, to be assumed again when the temperature falls. To leave it off on a cold day because you are going merely a short distance, is as careless as to stand talking with a departing guest on the front porch without your hat. In short, when worn it must be with judgment. Carelessness is always fruitful of disaster. Another question of importance is the presence of any chronic disease of the nose or throat. We find that people so afflicted are, as a general rule, subject to acute attacks even from very slight exposure. It is, therefore, a measure of prevention to treat any chronic disease of these parts. Of course, a complete discussion of what this treatment should be is not within the province of this paper. Attention, however, is earnestly directed to this subject, for I believe repeated acute attacks of coryza are merely symptomatic of some chronic fault, to which our investigations should be directed. To be sure, this supposes that ordinary care has been taken to avoid any exposure liable to develop the acute form of the disease.

If now, from neglect of all precaution, or in spite of the greatest care, a cold is developed, how shall it be treated? This brings us to the second part of the paper, viz.: Remedial measures. If the cold comes on towards evening, a hot bath should be taken, followed by the exhibition of 1-100 to 1-60 of a grain of sulphate of atropia, and the patient well wrapped up in bed. A word of caution is necessary in regard to the use of the atropia. Some people are very susceptible to its influence, and when given for the first time a much smaller dose must be used—say 1-250 to 1-200 of a grain. When found to disagree, a full dose of the sulphate of quinine, about ten grains, may be substituted. During the next day the nose and upper part of throat should be thoroughly washed out with a warm alkaline spray; or, still better, with a douche, by means of the posterior nasal syringe; the turgescence of the nasal tissues contracted by means of cocaine; and, finally, the entire surface covered with coating of an unirritating oil. The *oleum petrolina* we have found to be the best for this purpose. This should be done about three times in the course of the day. If the cold is first noticed in the morning, the process should be reversed; the local treatment given during the day and the general treatment at night. From experience we are able to report that, by this method, colds that generally last from ten days to two weeks can be limited to about two days. The discomfort after that is so very slight as not to be considered. We do not propose this as an infallible method, but where it fails it will usually be found that there is some intranasal chronic disease, or else some constitutional disorder which makes the attack of extraordinary obstinacy.

Lately, and while this paper was preparing, Dr. F. Cardone,[5] of Naples, has contended for the parasitic nature of acute coryza, stating that its morbid process is analogous to that of pneumonia. He has found in the secretions the *streplococcus pyogenes*, the *staphylococcus aureus et albus*, and, in still greater numbers, the *diplococcus* of Fraenkel and the *pneumococcus* of Friedlander. If he is correct, we can, perhaps, understand the great benefit resulting from the early and continued cleaning of the parts, such as the above treatment advocates. On the other hand, if a cold is caused by the paresis of the nerve-centers governing the vaso-motor phenomena of the upper air-tract, we can also understand how the counter-shock of the hot bath and atropia treatment, together with local depletion, so rapidly subdues the inflammatory action and restores the patient to accustomed health.

273 FRANKLIN STREET.

---

The Philadelphia Polyclinic has established a three months' systematic course in ophthalmology, particularly arranged to meet the needs of physicians who design to pay some special attention to ophthalmic practice. The course is largely clinical, but includes systematic didactic instruction and quizzing.

---

(5) Archio Italiani de Laringologia, Luglio, 1888.

